# Report of Deaths in the City of Buffalo, for the Month of August, 1862

**Published:** 1862-11

**Authors:** Sandford Eastman

**Affiliations:** Health Physician


*Report of Deaths in the City of Buffalo, for the month of August,* 1862.

Abscess 1, Accident 4, Accident by drowning 7, Apoplexy 2, Brain, softening of, 1, Cancer 1, Cholera infantum 27, Cholera morbus 3, Consumption 16, Convulsions 23, Cyanosis 1, Debility 1, Delirium tremens 2, Diabetes melitus 2. Diarrhoea 10, Disease of the brain 1, Disease of the heart 1, Disease of the spine 1, Diphtheria 1. Dropsy, general, 2, Dysentery 2, Epilepsy 1, Fever 3, Fever, puerperal 1, Fever, scarlet 10, Fever, typhoid 5, Fever, typhus 1, Hæmorrhage from uterus 1, Inflammation of bowels 1, Inflammation of brain 4, Inflammation and meninges 16, Inflammation of the liver 2, Inflammation of the lungs 2, Inflammation of the lungs, typhoid 1, Inflammation of the stomach 1, Intemperance 2, Jaundice 1, Kidneys, Bright's disease of 1, Marasmus 8, Measles 6, Neglect 1, Premature birth 2, Purpura hemorrhagica 1, Pyaemia 1, Scrofula 1, Syphilis 2, Unknown 5, Whooping cough 1. Total, 220. In addition to the above, 5 still-born were reported in this City.

*By whom certified.*—By regular physicians at public institutions, 15; by regular physicians in city at large, 97; by irregular practitioners, 43; coroner, 10; undertaker, 60. SANDFORD EASTMAN, Health Physician.

*Report of Deaths in the City of Buffalo for the month of Sept.* 1862.

